# Nilotinib versus imatinib with early switch from imatinib to nilotinib to obtain treatment-free remission in newly diagnosed chronic myeloid leukemia patients: the analysis of the first co-primary endpoint

**DOI:** 10.1038/s41375-025-02796-z

**Published:** 2025-11-18

**Authors:** Fausto Castagnetti, Massimo Breccia, Elisabetta Abruzzese, Renato Bassan, Gianni Binotto, Massimiliano Bonifacio, Giovanni Caocci, Isabella Capodanno, Francesco Cavazzini, Giuseppe Cimino, Paola Fazi, Antonella Gozzini, Alessandra Iurlo, Jeroen J. W. M. Janssen, Monia Lunghi, Roberto Marasca, Bruno Martino, Monica Messina, Francesco Muriano, Francesca Paoloni, Alfonso Piciocchi, Gianantonio Rosti, Antonella Russo Rossi, Giuseppe Saglio, Simona Sica, Simona Soverini, Agostino Tafuri, Daniele Vallisa, Peter E. Westerweel, Fabrizio Pane

**Affiliations:** 1 Department of Medical and Surgical Sciences, Institute of Hematology “Seràgnoli”, IRCCS Azienda Ospedaliero-Universitaria di Bologna, University of Bologna, Bologna, Italy; 2 Department of Translational and Precision Medicine, Hematology Sapienza University, Rome, Italy; 3 Hematology, S. Eugenio Hospital, Tor Vergata University, Rome, Italy; 4 Division of Hematology, Ospedale dell’Angelo, Mestre-Venice, Italy; 5 Department of Medicine, Hematology Unit, University of Padova, Padova, Italy; 6 Department of Engineering for Innovation Medicine, Section of Innovation BIomedicine, Hematology Area - University of Verona, Verona, Italy; 7 Department of Medical Sciences and Public Health, Businco Hospital, University of Cagliari, Cagliari, Italy; 8 SOC Ematologia Azienda USL-IRCSS di Reggio Emilia, Reggio Emilia, Italy; 9 Division of Hematology, University of Ferrara, Ferrara, Italy; 10 Department of Translation and Precision Medicine, University “La Sapienza”, Rome, Italy; 11 GIMEMA Foundation, Data Center and Health Outcomes Research Unit, Rome, Italy; 12 Hematology Unit, AOU Careggi, University of Florence, Florence, Italy; 13 Hematology Division, Foundation IRCCS Ca’ Granda Ospedale Maggiore Policlinico, Milan, Italy; 14 Department of Hematology, Radboud University Medical Center, Nijmegen, The Netherlands; 15 Division of Hematology, Department of Translational Medicine, AOU Maggiore della Carità, Università del Piemonte Orientale, Novara, Italy; 16 Hematology Unit, Department of Medical and Surgical Sciences, Azienda Ospedaliera Universitaria di Modena, University of Modena and Reggio Emilia, Modena, Italy; 17 Hematology Unit, Grande Ospedale Metropolitano “Bianchi-Melacrino-Morelli”, Reggio Calabria, Italy; 18 Department of Clinical Medicine and Surgery, Hematology Section, University of Naples “Federico II”, Naples, Italy; 19 GIMEMA Data Center, Rome, Italy; 20 Medical Oncology Unit, IRST/IRCCS “Dino Amadori”, Meldola, Italy; 21 Hematology and Stem Cell Transplantation Unit, AOU Consorziale Policlinico, Bari, Italy; 22 Department of Clinical and Biological Sciences, University of Turin, Turin, Italy; 23 Haematology Unit, Department of Radiological and Hematological Sciences, Università Cattolica del Sacro Cuore, Rome, Italy; 24 Department of Medical and Surgical Sciences, Institute of Hematology “Seràgnoli”, University of Bologna, Bologna, Italy; 25 Department of Clinical and Molecular Medicine and Hematology, Sant’Andrea - University Hospital - Sapienza, University of Rome, Rome, Italy; 26 Civic Hospital of Piacenza, Piacenza, Italy; 27 Department of Internal Medicine, Albert Schweitzer Hospital, Dordrecht, The Netherlands; 28 Department of Oncology and Hematology, University Federico II of Naples, Naples, Italy

**Keywords:** Chronic myeloid leukaemia, Randomized controlled trials

## Abstract

Treatment-free remission is one of the most important goals of CML treatment but so far, the best treatment to reach this aim is still undefined, even though it is widely accepted that a sustained DMR is the prerequisite to discontinue TKI. Here we report on the depth of the molecular response, the first co-primary end point of the SUSTRENIM study, in a cohort of newly diagnosed CP-CML patients randomized 1:1 to be treated with nilotinib or with imatinib followed by switching to nilotinib in absence of optimal response. Of the 448 enrolled patients, 228 and 220 were randomized to the nilotinib (NIL) and imatinib (IM) arms, respectively, and followed for a median of 45.9 months. Eighty-two (37.2%) of the 220 patients on the IMarm did not fulfill the ELN criteria for optimal response of treatment and switched to nilotinib therapy. At the 24 months of follow-up, 107 of the 448 patients reached an MR4.5 response with a significantly higher frequency within the patients on the nilotinib arm (65 vs 42; *p* = 0.02). The analysis of the first primary endpoint indicates that, despite the early switch in the IM-randomized patients, NIL therapy is more effective to induce DMR.

## Introduction

Nowadays, in the era of tyrosine kinase inhibitors (TKI), an important final endpoint in the management of chronic phase chronic myeloid leukemia (CP-CML) patients is the achievement of a sustained and deep molecular response (DMR) for subsequent discontinuation, namely a treatment-free remission (TFR), defined as a sustained leukemic mass reduction induced by therapy followed by treatment discontinuation without the need to regain the therapy [1–3].

The choice of the first-line TKI when the aim of CML therapy is TFR has not yet been settled, and is a matter of debate, particularly in elderly patients with comorbidities in which the therapy with a most potent second-generation TKIs might be associated with mid- or long-term off-target effects [1, 3–5]. We barely know the percentage of patients who start treatment and ultimately reach this goal [6]. In addition, we do not have any reliable prognostic markers at baseline to predict TFR, except the long treatment duration of TKIs and the median duration of DMR [7]. In fact, data from several important, but small and uncontrolled, retrospective studies, have provided proof of principle that a median duration of TKIs for more than 5 years can select patients for TFR and that an attempt to discontinuation can be considered in cases of DMR long-lasting for more than 3 years in case of MR4 (BCR::ABL1 < 0.01%IS) or more than 2 years for MR4.5 (BCR::ABL1 < 0.0032%IS) [6–12]. However, so far, the best strategy to reach TFR is still undefined. Second generation TKIs, being more potent and selective on *BCR::ABL1*, can induce a more rapid and deep reduction of the disease burden compared to Imatinib (IMA), and therefore might represent the baseline treatment of choice for a final TFR [13–15]. These drugs allow a rapid decline of the disease burden during the first three months of therapy, the so-called early molecular response (EMR), which well correlates with the probability to achieve a stable and sustained DMR, the most important known prerequisite to discontinue TKI [16, 17]. However, the potential long-term toxicity observed with TKI-specific off-target effects of the second generation TKIs usually reduces their use as frontline treatment in specific subgroups of patients, such as the elderly and those affected by cardiac, vascular or lung disorders and hence limits their possibility to attempt to a successful TFR [18, 19]. In these patients, the control of the disease over time is nowadays a more realistic and conservative approach that, therefore, is modulated to achieve the best balance between optimal control of the disease and the toxicity of therapy. In this context, the identification of those patients whose disease characteristics preclude the possibility of TFR is a very important aim of future investigations.

An intriguing possibility to increase the overall TFR probability is evaluating the possibility of an early switch from IM to a second-generation TKI only in those patients who fail to achieve the EMR, i.e., in those with the smallest probability to achieve the DMR and a successful TFR, sparing the potential toxicity of the second-generation TKIs in the others.

In this background, the SUSTRENIM study is the first and, so far, the unique prospective study comparing not only the rate of DMR but, more importantly, also the rate of TFR according to treatment: a second-generation TKI frontline vs IM frontline followed by systematic switching to the same second-generation TKI in case of non-optimal response.

Here we show the definitive results of the first co-primary endpoint of this trial, i.e., the pre-planned interim analysis on the rate of DMR at 24 months.

## Materials and methods

The SUSTRENIM trial (Clinicaltrias.gov NCT02602314) is a prospective, interventional, randomized, two-arm study in newly diagnosed CP-CML patients treated with a second generation TKI (Nilotinib, NIL) or with IM followed by switching to NIL in absence of optimal response. The trial started in November 2016 and involved 53 Italian and 8 Dutch centers. Patients are randomized 1:1 between NIL and IM according to Sokal risk score (high versus intermediate/low risk) and country, However, the ELTS score was calculated for each patient enrolled in the study as an important baseline characteristic to correlate with the primary endpoints.

The main exclusion criteria were uncontrolled serious medical conditions, prior treatment with whatever TKI and the expression of any atypical *BCR::ABL1* transcripts. Patients will be treated according to the registered dose of NIL and IM for frontline chronic phase CML (300 mg BID and 400 mg OAD, respectively) and monitored for residual disease at a three-monthly interval. Molecular response will be assessed at a local network of standardized laboratories in all patients by RQ-PCR testing of *BCR::ABL1* levels in peripheral blood. The results, converted to International Scale (IS), were classified as MMR, MR4.0, and MR4.5 according to the standardized definitions of MR proposed by the European LeukemiaNet in its most recent recommendations for treatment of CML patients available at that time [1, 20]. All the patients who were intolerant to IM and the patients who missed the criteria qualifying for optimal response at 3, 6, and 12 months, as defined in the ELN 2013 recommendations [21] were switched to 300 mg BID NIL therapy except for the patients with progression to accelerated or blast phase who went off study. All the patients who obtained a reduction greater than 4.0 logs of residual disease (MR4.0) within the first three years of treatment and maintain this level of response in all the 4 subsequent tests up to the end of the fourth year of therapy qualified, in the second part of this trial, for the discontinuation phase of the study.

The whole study had two sequential co-primary endpoints: (1) the rate of molecular response (MR4.5) at 24 months, reported in this manuscript and (2) the rate of patients remaining in prolonged TFR (≥MMR) without molecular relapse, 12 months after entering the TFR phase.

Statistical analysis has compared the two randomized treatment groups under the Intention-to-Treat (ITT) principle. A total of 450 patients (225 per treatment group) were planned to be enrolled in the study to guarantee the robustness of the statistical analysis.

A detailed study protocol and the sample size computation for the two co-primary endpoints are shown in the supplementary materials of this manuscript.

## Results

### Patient features at baseline

**Fig. 1:**
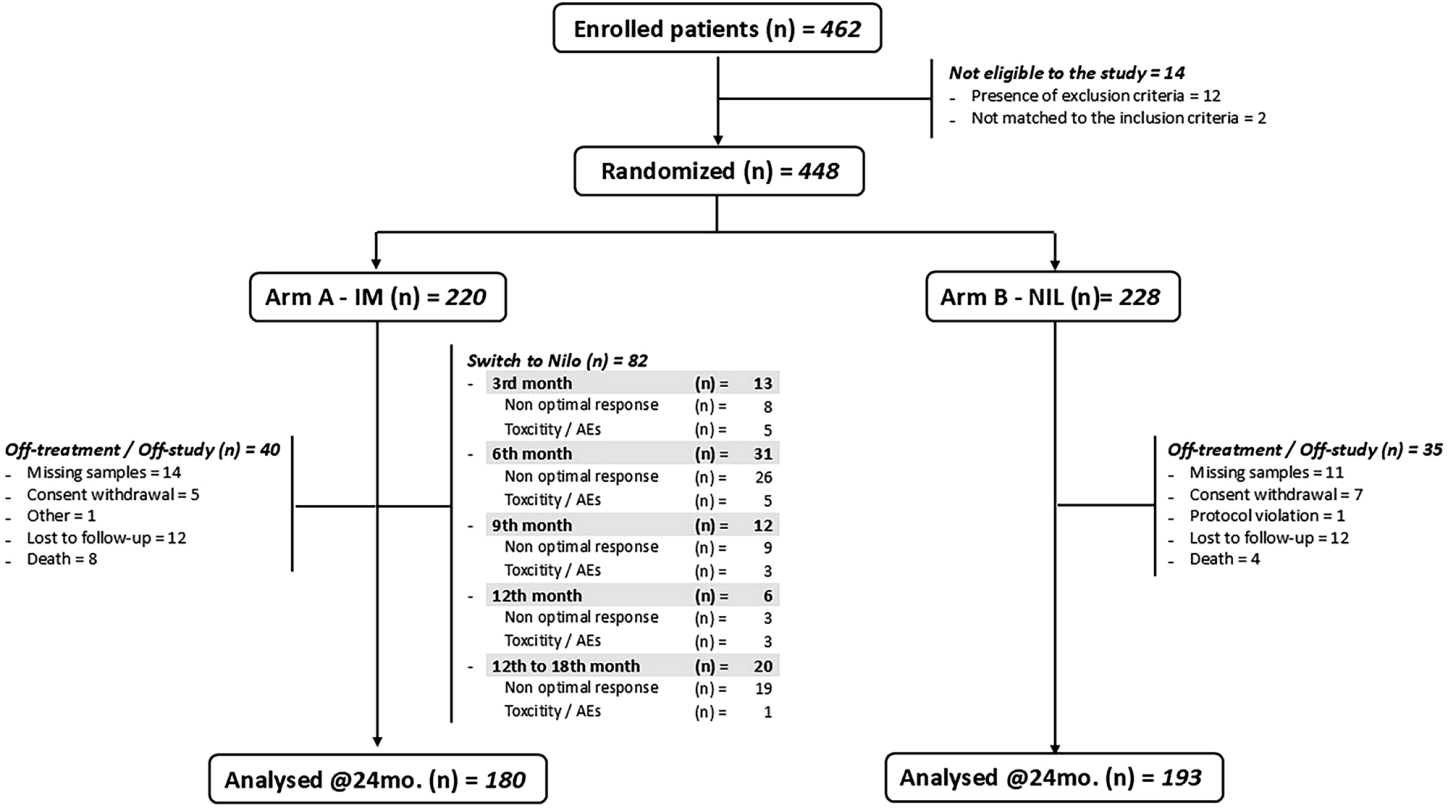
Patient disposition at the last follow-up.

In the period between November 2016 and January 2021, 448 of the 462 patients consecutively enrolled in the study were randomized to the NIL (*N *= 228) and IM (*N* = 220) arms of the study, while the remaining 14 were excluded as they did not match the inclusion criteria (*n* = 2) or because they showed one or more exclusion criteria (*n* = 12) (Fig. 1). The main features of these patients at disease onset were well balanced after randomization between the two study arms (Table 1). Overall, 258 patients (58%) were males and 190 (42%) were females, their median age at diagnosis was 54 years (range, 19–86) and 20% and 28% were aged >65 years in the NIL and IM arms, respectively. Overall, the percentage of high-risk patients was 16% according to the Sokal score and 9% for the ELTS score [22] (Table 1). At baseline, evaluable cytogenetic analysis was detected in 360 out of 448 patients. The vast majority of the patients (*n* = 335) showed a classical t(9:22) translocation while in 15 patients, a complex translocation was detected that gave origin to the *BCR::ABL1* transcript. As expected in patients analyzed at baseline, the rate of additional chromosomal aberrations (ACA) was low with only 10 (2.2%) patients observed, classified as low risk in 3 cases and high-risk ACA in the remaining 7 (Table 1).

**Table 1:** Patients’ demographic and baseline features.

Characteristic	Overall, *N* = 448	Nilotinib arm, *N* = 228	Imatinib arm, *N* = 220
Age, median (years, range)	54 (19, 86)	52 (19, 86)	56 (20, 84)
Age in classes, *n* (%)			
*18–65 years*	341 (76%)	183 (80%)	158 (72%)
*>65 years*	107 (24%)	45 (20%)	62 (28%)
Sex, *n* (%)			
*Male*	258 (58%)	127 (56%)	131 (60%)
*Female*	190 (42%)	101 (44%)	89 (40%)
Sokal score, *n* (%)			
*Low*	183 (41%)	102 (45%)	81 (37%)
*Intermediate*	191 (43%)	90 (39%)	101 (46%)
*High*	72 (16%)	36 (16%)	36 (17%)
*Unknown*	2	0	2
ELTS score, *n* (%)			
*Low*	278 (62%)	151 (66%)	127 (58%)
*Intermediate*	128 (29%)	58 (25%)	70 (32%)
*High*	40 (9.0%)	19 (8.3%)	21 (9.6%)
*Unknown*	2	0	2
Additional aberrations in the Ph clone, *n* (%)			
*No additional aberration*	335 (74.7%)	180 (78.9%)	155 (70,4%)
*Complex Ph translocations*	15 (3.3%)	8 (3.5%)	7 (3.1%)
*ACA*	10 (2.2%)	5 (2.1%)	5 (2.3%)
*Unknown*	88	35	53

*ELTS* EUTOS long term survival score, *ACA* Additional chromosomal abnormalities.

### Switch to nilotinib arm

Overall, 82 of the 220 patients randomized to the IM arm (37.2%) did not achieve the optimal response criteria according to ELN definitions (*n* = 65) or showed poor tolerance to therapy (*n* = 17) and, according to the protocol design, were switched to NIL therapy at 300 mg BID. In 61 patients (74%), treatment switch was done within the first 12 months of therapy while in the remaining 21 cases (26%) treatment was changed during the subsequent 6 months of the study follow-up. Figure 1 shows the detailed times and causes of patient switching to NIL. The age of all these patients did not differ significantly from those who achieved optimal response milestones and remained on IM therapy (Table 2). Similarly, neither gender nor the presence of P190 encoding BCR::ABL1 transcript had any effect on the treatment switching probability (Table 2). On the other hand, high risk Sokal and even more the high ELTS score, significantly impact on IM to NIL therapy switch rate. Indeed, 19 of the 36 patients (52.8%) at high Sokal risk (*p* = 0.05) and 13 of the 21 patients (61.9%) at high ELTS risk patients (*p* = 0.01) of the IM arm were moved to NIL within the first 24 months of therapy (Table 2).

**Table 2:** Features of the patients of the IMA arm (*n* = 220) who switched to NIL therapy due to non-optimal response to the therapy.

Characteristic	Overall *N* = 220	Not switched *N* = 138	Early switch (<12 months) *N* = 61	Late switch (>12 months) *N* = 21	*p*-value^a^
Age, years, median (range)	56 (20, 84)	59 (20, 84)	53 (22, 81)	52 (20, 77)	**0.050**
Age in classes, *n* (%)					0.56
*18–65 years*	158	96 (60.8%)	45 (28.5%)	17 (10.8%)	
*>65 years*	62	42 (67.7%)	16 (25.8%)	4 (6.5%)	
Sex, *n* (%)					0.30
*Male*	131	84 (64.1%)	32 (24.4%)	15 (11.5%)	
*Female*	89	54 (60.7%)	29 (32.6%)	6 (6.7%)	
Sokal score, *n* (%)					0.12
*Low*	81	54 (66.7%)	17 (21.0%)	10 (12.3%)	
*Intermediate*	101	67 (66.3%)	27 (26.7%)	7 (6.9%)	
*High*	36	17 (47.2%)	15 (41.7%)	4 (11.1%)	
*Unknown*	2	0	2	0	
ELTS risk, *n* (%)					**0.011**
*Low*	127	91 (71.7%)	28 (22.0%)	8 (6.3%)	
*Intermediate*	70	39 (55.7%)	21 (30.0%)	10 (14.3%)	
*High*	21	8 (38.1%)	10 (47.6%)	3 (14.3%)	
*Unknown*	2	0	2	0	

^a^Kruskal–Wallis rank sum test; Fisher’s exact test.

*ELTS* EUTOS long term survival score.

Bold values indicate statistical significance *p* ≤ 0.05.

### Follow-up and rate of MR4.5 at 24 months

At the time of this analysis of the first co-primary endpoint of the study, the median follow-up of the entire cohort of patients enrolled in the study was 45.9 months (range 1.7–73) and all patients had at least 24 months of follow-up. The analysis was carried out using the Fisher’s exact test under the intention to treat principle. After 24 months of follow-up, 75 patients, 40 of the IM arm and 35 of the NIL arm, were off-study or off-therapy and considered as failures, as shown in Fig. 1. Overall, 12 patients died, 8 of the IM and the remaining 4 of the NIL arms. In only one case, death was due to disease progression to blast crisis (IM arm), in 3 cases for cardiac or vascular reasons, and in the remaining 8 patients for causes unrelated to the disease or treatment such as second primary malignant neoplasia. In 18 patients (2.6%, 12 of the NIL arm and 6 of the IM arm), this was due to treatment failure and moved to other therapies, while in the remaining the off study was due to consent withdrawal or visit or molecular analysis not done at that point. All those patients were considered failures in the statistical analysis.

Overall, MR4.5 response was reached by 107 of the 448 randomized patients (24%) with a statistically significant prevalence of patients of the NIL arm vs IM arm despite treatment early optimization of those latter (29% vs 19%, *p* = 0.02) (Table 3).

**Table 3:** Comparison of MR4.5 rates at 24 months of follow-up between the two randomization arms based on an intention-to-treat analysis.

	No. of pts	No MR4.5		MR4.5		Fisher’s exact test
		*n*	*%*	*n*	*%*	
Imatinib arm	220	178	71%	42	19%	*P* = 0.02
Nilotinib arm	228	163	81%	65	29%	
Total randomized	448			107	24%	

No MR4.5 = patients with MRD level higher than MR4.5, missing data or visit at 24 months and those who went off treatment due to AE, no response, progression, or death.

It is noteworthy that switching treatment does not appear to be sufficiently effective in bridging the gap between the mean depth of response at 24 months of patients randomized to the IM arm versus those of patients in the NIL arm. In fact, 28 of the 116 patients (24%) who had an optimal early response to IM treatment and, according to the study protocol, were still on therapy at 24 months, achieved MR4.5, while this response was achieved in only 10 of 58 (17%) and 4 of 20 (20%) patients who switched to NIL within the first 12 months (early switch) or later in the following 12 months (late switch, Table 4).

**Table 4:** MR4.5 rates at 24 months of follow-up in patients randomized to IM arm according to early or late switch to NIL therapy.

			Switch to NIL		
MR at 24 months	Overall *N* = 194	No switch *N* = 116	Early <12 months *N* = 58	Late >12 months *N* = 20	*p*-value^a^
Less than MR4.5, *n* (%)	152 (78%)	88 (76%)	48 (83%)	16 (80%)	0.59
MR4.5 or better, *n* (%)	42 (22%)	28 (24%)	10 (17%)	4 (20%)	

^a^Fisher’s exact test.

**Fig. 2:**
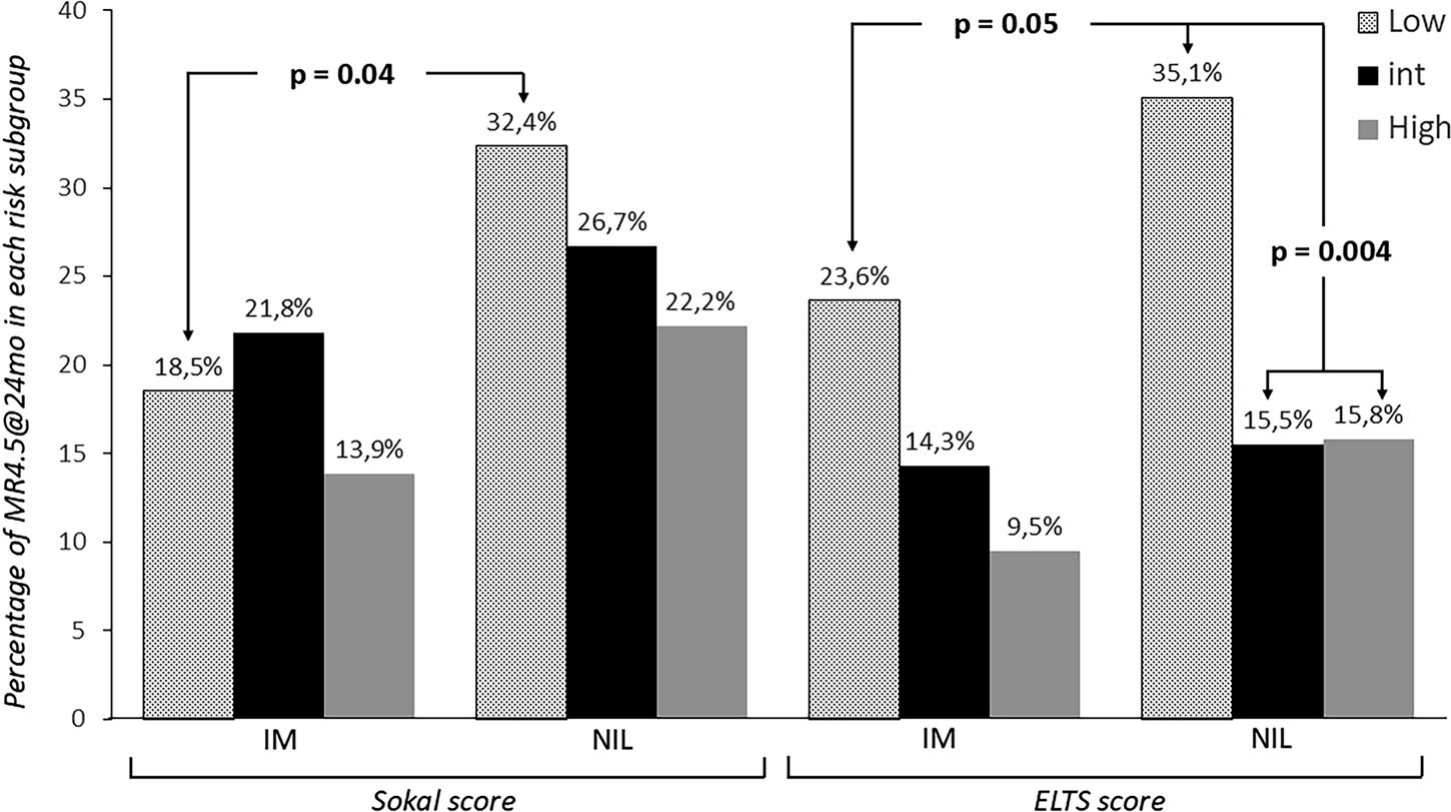
Rate of MR4.5 at 24 months of follow-up according to either Sokal or ELTS risk scores in patients stratified per treatment arm.

If the treatment arm is the strongest factor to predict the probability of DMR (MR4.5) at 24 months of treatment, the risk score significantly correlates to the probability rate of MR4.5 at 24 months of treatment, but only when the ELTS stratification has been used. Table 5 shows that the ELTS, but not the Sokal score, together with the randomization arm hold statistically significant correlation to MR4.5 rate at multivariate analysis, while neither gender nor age were able to predict deep molecular remission. Indeed, when the Sokal risk stratification has been considered, we only found a statistical difference in the rate of MR4.5 at 24 months between the patients at low risk who receive frontline NIL therapy as compared to those randomized to the IM-optimized treatment (32.45 vs 18.5%, *p* = 0.04, Fig. 2). On the other hand, ELTS stratification has been shown to be able to stratify the probability rate of MR4.5 in patients randomized to both the NIL and IM arms with an odds ratio of 0.33 in intermediate-risk and high-risk patients compared patients are at low risk (Fig. 2 and Table 5). Interestingly, 35.1% of low ELTS risk patients randomized to NIL frontline therapy and 23.6% of the same subgroup randomized to IM with early optimization achieve MR4.5 at 24 months (*p* = 0.05), and hence, the former appears to be those who most benefit of NIL treatment frontline as compared to all the others. Therefore, being a low risk at baseline according to the ELTS stratification, predicts the highest rate of MR4.5 at 24 months of treatment (Fig. 2). Among the patients at low Sokal risk, randomization to receive NIL front line showed a significant superiority respect to IM randomization in terms of probability to reach a DMR (Fig. 2 and Table 5).

**Table 5:** Odds ratios of achieving MR4.5 at 24 months of follow up in multivariate analysis in patients stratified by Sokal or ELTS risk scores.

Sokal score stratification				ELTS score stratification			
	OR^a^	95% CI^a^	*p*-value		OR^a^	95% CI^a^	*p*-value
Treatment arm				Treatment arm			
*Nilotinib*	—	—		*Nilotinib*	—	—	
*Imatinib*	0.59	0.37, 0.93	**0.025**	*Imatinib*	0.61	0.38, 0.97	**0.040**
Age	1.00	0.99, 1.02	0.74	Age	1.01	1.00, 1.03	0.12
Sex				Sex			
*Male*	—	—		*Male*	—	—	
*Female*	1.12	0.71, 1.77	0.62	*Female*	1.00	0.63, 1.59	>0.99
Sokal score				ELTS score			
*Low*	—	—		*Low*	—	—	
*Intermediate*	0.88	0.51, 1.49	0.63	*Intermediate/High*	0.33	0.19, 0.57	**<0.001**
*High*	0.57	0.27, 1.15	0.13				

^a^*OR* odds ratio, *CI* confidence interval.

*ELTS* EUTOS long term survival score.

Bold values indicate statistical significance *p* ≤ 0.05.

Overall, 97 patients had more than 65 years at enrollment into the study and of those, 42 were randomized to the NIL while the remaining 55 to the IM arm. Interestingly, 16 of the former group and only 10 of the latter achieve MR4.5 (38% vs 18%, *p* = 0.05), showing that NIL therapy is feasible also in advanced age.

### Adverse events

Table 6 shows that the adverse events considered related to the therapy recorded in the patients enrolled into the study. Overall, the adverse events were 276 in the patients of the NIL arm vs 352 in the patients initially randomized to the IM arm. However, the adverse events counted in these latter cases also include those 32 that occurred in the 82 patients who switched to NIL after changing therapy. On the whole, at least one adverse event has been observed in 129 of the 220 patients (58.6%) randomized to the IM arm, and in 148 of the 228 patients (64.9%) randomized to the NIL arm. The most common adverse events, the cytopenia, and those occurring at musculoskeletal, gastrointestinal, and ocular levels are more frequent in the patients randomized to the IM arm, while those occurring at the skin or at the nervous system were more frequently reported among the patients randomized to the NIL arm and more generally in those assuming NIL. Noteworthy, grade 3 or 4 events were rather infrequent and well balanced between the two arms of the study (29 in the IM arm vs 30 in the NIL arm).

**Table 6:** Adverse reactions subdivided in columns according to the randomization arm and stratified according to the system and organ classes.

	IM arm (*n* = 220)					IM arm switched to NIL (*n* = 82)					NIL arm (*n* = 228)					
	Grade 1	Grade 2	Grade 3	Grade 4	Total	Grade 1	Grade 2	Grade 3	Grade 4	Total	Grade 1	Grade 2	Grade 3	Grade 4	Total	Total (All patients)
Blood and lymphatic system	17	12	10	2	**41**			1		**1**	8	7	5	2	**22**	64
Skin and subcutaneous tissue	24	9	3		**36**	10	2			**12**	48	25	3		**76**	124
Musculoskeletal and connective tissue	53	17	3		**73**	1	1	1		**3**	22	7			**29**	105
Gastrointestinal	38	17	2		**57**	3	2	1		**6**	20	6	2		**28**	91
Systemic	38	8	4		**50**	1				**1**	22	14			**36**	87
Ocular	36	7			**43**					**0**	6	3	3		**12**	55
Nervous system	7				**7**	1	1			**2**	7	4	2	1	**14**	23
Metabolism and nutrition	2	2			**4**		1			**1**	10	4	1		**15**	20
Infections	2	1			**3**	1	1			**2**	4	5			**9**	14
Hepatobiliary	1				**1**					**0**	2	3	6		**11**	12
Vascular	1				**1**		1	1		**2**	1	4	1	1	**7**	10
Cardiac			1		**1**			1		**1**	1	3	1	1	**6**	8
Respiratory thoracic and mediastinal		1			**1**							3			**3**	4
Psychiatric		1			**1**	1				**1**	1				**1**	3
Reproductive system					**0**					**0**	2		1		**3**	3
Endocrine					**0**					**0**	1	1			**2**	2
Renal and urinary					**0**					**0**	2				**2**	2
Neoplasms			1		**1**					**0**					**0**	1
Total	**219**	**75**	**24**	**2**	**320**	**18**	**9**	**5**		**32**	**157**	**89**	**26**	**5**	**276**	**628**

After the hematopoietic adverse events, the other classes are listed in order of occurrence frequency. Adverse events occurred after treatment switching to Nilo are listed in a separate section of the table.

Bold values indicate statistical significance *p* ≤ 0.05.

As at least partly expected, either vascular and cardiac events were more frequently observed in patients randomized to NIL arm or switched to receive NIL. Indeed, only one single episode of grade 1 arterial stenosis has been recorded in a patient while receiving IM, whereas 7 clinically significant arterial stenosis events (two of grade 3 and one grade 4 stroke), one case of therapy related arterial hypertension and one of retinal vasculitis have been recorded in patients while receiving NIL. Also, cardiac adverse events were more frequent in patients receiving NIL therapy. Only one case of grade 3 atrioventricular block occurred under IM treatment, while 7 cardiac adverse events were reported in patient randomized or switched to NIL therapy. Among those latter, two grade 3 events and one grade 4 cardiac infarction. Two deaths due to cardiac infarction have been recorded, one under IM and the second under NIL therapy. Both were not clearly related to the drug were receiving by the patients. In addition, only 1 death occurred due to vascular cause in a patient under IM therapy for cerebral hemorrhage.

Finally, we did not observe significant differences between the type and rate of adverse events in younger versus older patients. This finding is supported by the results of the multivariate analysis of prognostic factors related to the rate of MR4.5 at 24 months (Table 5), which excluded any response bias due to patient age.

## Discussion

The choice of the first-line TKI when the aim of CML therapy is TFR has not yet been settled, and is a matter of debate, particularly in elderly patients with comorbidities in which the therapy with a most potent second-generation TKIs might be associated with mid- or long-term off-target effects [1, 3–5]. This trial involved 61 academic clinical centers with clinical experience in the management of CML and compares in a prospective and randomized approach two strategies of front-line therapy of CML with the final aim of discontinuation instead of overall survival. In this trial, for the first time, the efficacy of the therapy with NIL has been compared to IM therapy with early switch to NIL in cases of non-optimal response according to the definitions of ELN 2013 recommendations [21] (but still unchanged in the 2020 ELN edition). Indeed, while switching treatment in case of failure is considered mandatory both in ELN recommendations and NCCN guidelines [1, 3], less clear is what to do in the more frequent cases of responses classifiable as “warning” or “not optimal”. This is particularly true for those patients in which the aim of therapy is TFR, and until now, the unique suggestion for patients with non-optimal response in the first months of therapy is a closer monitoring of the molecular residual disease (MRD). Here, we report the efficacy in terms of DMR, i.e., MR4.5 rate at 24 months of systematic switching to second-generation TKIs in patients treated front line with IM in case of suboptimal response during the first 12 months of treatment. The study is based on the rationale that timely switching of TKIs can improve the depth of the molecular response, thus preserving the long-term probability of TFR, while avoiding those patients with an optimal initial response to IM from potential and unnecessary additional toxicity. In this regard, it should be noted that treatment intensification, i.e., increasing the IM dose or switching from IM to NIL, was adopted in the TIDEL II study, a clinical trial published by the Australasian Leukemia and Lymphoma Group. The study concluded that this strategy may be advantageous in terms of depth of molecular study or survival when applied to patients with low serum imatinib levels, unsatisfactory molecular responses, or poor tolerance to therapy [23].

In our study, 37% of patients who started IM therapy at the standard dose of 400 mg switched to NIL therapy, primarily in the first 12 months, due to a suboptimal molecular response (*n* = 65) or poor treatment tolerance (*n* = 17) without any advantage in those who switched therapy in the first 12 months of treatment compared to those who switched therapy later in the subsequent 6 months, although the significance of the comparison may be weakened by the fact that the latter may belong to a more favorable response group, having already achieved all early optimal molecular response objectives. It is important to note that only ELTS risk score [22] and not the other clinical or demographic features canonically recorded at baseline significantly correlated to the probability of non-optimal response. Indeed, 13 of the 21 patients with high ELTS score in the IM arm (61.9%) switched to NIL while only 36 of the 127 patients at low ELTS risk (28.3%) were moved to NIL therapy.

Remarkably, the comparison of the rates of MR4.5 at 24 months of therapy, i.e., the primary co-end point of this trial, is significantly in favor to the NIL arm of the trial (29% vs 19%) despite a total of 37% of patients of the IM arm switched very early to NIL. Therefore, beginning therapy with NIL guarantees the highest probability of early DMR, and at least in principle the best chances of sustained deep molecular remission and of a successful TFR. However, it is worth to note that the value the treatment switch timely applied upon missing the milestones indicating optimal response, is proven by the observation that, while the rate of MR4.5 in the patients of NIL arm is little higher than that expected compared with the data of the ENESTnd trial [24], those of IM arm are much higher than those expected with IM frontline without any treatment switch.

In this trial we also investigated the prognostic role of the deep response: in multivariate analysis, if we consider that the treatment arm is the strongest predictor of the probability to reach rapidly DMR, a very good response was observed in the low ELTS risk patients. In fact, when treated with NIL frontline, 35% of the low ELTS risk patients vs only 15% of those with intermediate or high ELTS risk reached this important milestone. This result is not surprising considering the effect of age as lower impact in ELTS than in the Sokal score. Consequently, the Sokal score classifies inappropriately more elderly patients (24% in this trial) than ELTS in the intermediate- and high-risk groups, and this is not useful to plan an intensification of treatment.

Our trial enrolled an unselected cohort of treatment naïve patients with no age limits with the only exclusion of patients with poorly controlled cardiovascular disorders. Therefore, a physiologically high number of elderly patients were included in the two cohorts of patients that showed an overall median age of 54 years and 24% of patients older than 65 years. It is important to note that we did not observe any significant imbalance in the incidence of side effects either in patients randomized to the NIL arm or in those switched to NIL therapy due to suboptimal response, or in older patients >65 years of age. Nevertheless, the multivariate analysis did not show any detrimental effect of age on the probability of reaching an early DMR.

In the case of cardiac and vascular toxicity, the number of adverse events was higher in patients randomized to NIL and in those randomized to IM but switched to NIL (9 and 7 vascular and cardiac events, respectively) compared to those in patients treated with IM alone (1 and 1 vascular and cardiac event, respectively). However, we observed only two cardiac-related deaths, one in the patient treated with NIL and the other in the patient treated with IM.

In conclusion, this study demonstrates that, if the final goal is TFR, possibly to be achieved within a reasonable time from diagnosis, i.e., 4–6 years, an aggressive therapeutic strategy can be pursued in all patients. First-line therapy with second-generation TKIs is feasible and more effective to achieve a deep and EMR and, consequently, to be a candidate to achieve TFR compared to IM despite an optimization with early switch to NIL in case of suboptimal response. This appears to be particularly true in the case of low ELTS risk patients, regardless of their age, gender, or other baseline features. On the other hand, in patients at intermediate or high ELTS risk, the benefit of NIL frontline is negligible if the aim of the therapy is deep molecular remission and TFR achievement. Therefore, these patients may begin the treatment with IM optimizing with NIL only in case of early non-optimal response. In the near future, the evaluation of the results regarding the second co-primary endpoint of the study will show us whether the highest rates of early DTMR translate into the highest probability of successfully TFR.

## Supplementary information

Supplementary material

## Data Availability

The data sets support the results reported in this article are available at the GIMEMA Data Center. The data are provided in compliance with applicable laws, data protection, and requirements for consent and anonymization.
