# Supplementary material for: Nilotinib versus imatinib with early switch from imatinib to nilotinib to obtain treatment-free remission in newly diagnosed chronic myeloid leukemia patients: the analysis of the first co-primary endpoint
Source: Leukemia. 2025 Nov 18;40(1):47–54. doi: 10.1038/s41375-025-02796-z (PMC12789014; doi:10.1038/s41375-025-02796-z)

**Trial design**

This is a prospective, interventional, randomized, two arms, phase IV study evaluating both the depth of the molecular response and the rate of treatment free remission rate in newly diagnosed CP-CML patients treated with NIL or IM followed by switch to NIL in absence of optimal response (defined according the ELN 2013 criteria) as per clinical practice (*see also figure 1 of this MNS section*).

The enrolled patients have been randomized 1:1 between NIL and IM. Patients have been stratified according to the Sokal risk score to high versus intermediate/low risk groups and Country (Italy, The Netherlands, Belgium). Newly diagnosed patients have been treated according to the registered dose of NIL and IM for frontline chronic phase CML (300 mg BID and 400 mg OAD, respectively). The patients intolerant to IM and the patients with less than optimal response to IM at 3 months, at 6 months, at 12 months (except the patients with progression to accelerated or blastic phase) have been switched to NIL second line.

The absence of optimal response is defined by at least one of the following ELN criteria: a) Absence of Complete Hematologic Response at 3 months or thereafter; b) Absence of Partial Cytogenetic Response (> 35% Ph+ metaphases) at 3 months; c) BCR-ABL transcript level > 10% according to the IS at 3 months; d) Absence of Complete Cytogenetic Response (> 1% Ph+ metaphases) at 6 months; e) BCR-ABL transcript level > 1% according to the IS at 6 months; f) Absence of Major Molecular Response (MR3.0, transcript level > 0.1% according to the IS) at 12 months.

Treatment choice for the patients with progression to advanced disease phase while on IM and for the patients intolerant to or resistant (including progressions to advanced phases) to NIL will be up to the principal investigator of the participating Center. They are “Off-study”, however, information concerning the course and outcome of these patients will be collected and recorded for at least 5 years, and they could be enrolled in investigational studies promoted by GIMEMA or other sponsors.

After the induction of deep molecular remission phase of therapy, i.e. the first two years of the study, residual disease will be closely monitored (quarterly) by Q-PCR assays. All the patients who obtain a reduction greater than 4.0 logs of residual disease (MR4.0) within the first three years of treatment and maintain this level of response in all the subsequent tests up (Sustained MR4.0) to the end of the fourth years of therapy qualify for the discontinuation phase of the study. The sustained MR4.0 is thus defined in the study as 12-month period during which the MR4.0 never is lost in 4 consecutive MRD analyses at three-monthly intervals. Therefore, all patients who are in a sustained MR4.0 at the end of a four-year period of TKI treatment enter the treatment free remission (TFR) phase of the study. In case of loss of MR3.0, the last assumed TKI will be resumed at the same dose.

All patients, including those who do not match the criteria for discontinuation of TKI treatment, have been continued the assigned treatment and followed for 5 years, starting from the date of enrolment.

**Objectives of the study**

The study will investigate in newly diagnosed CP-CML patients the efficacy of NIL frontline therapy vs IM followed by switch to NIL in the case of absence of optimal response as defined by the ELN criteria using two primary endpoints:

− To evaluate the rates of molecular response (MR4.5) at 24 months

− To evaluate the rate of patients who remain in sustained treatment free remission (≥MR3.0) without molecular relapse 12 months after entering the TFR phase. The molecular relapse is defined as loss of MMR or confirmed loss of MR3.0.

**Secondary Objectives**

1. Monitoring the molecular response.

2. Progression-free survival (PFS) in the two arms of the study.

3. Overall Survival in the two arms of the study.

4. Rates of major molecular response (MR3.0) during the study in the two arms of the study.

5. The dynamics of molecular response along the whole study.

6. The relationship between baseline characteristics and the primary objectives.

7. The relationship between early molecular response and the primary objectives.

8. To assess the safety profile of both arms.

9. To determine the rate and the time-distribution of the discontinuation causes of the first-line TKI.

10. To investigate quality of life (QoL) differences between treatment arms over time.

**Study Population**

Newly diagnosed chronic phase BCR/ABL+ CML adult patients.

**Inclusion Criteria**

Patients eligible for inclusion in this study have to meet all of the following criteria:

- Patients with a confirmed diagnosis of BCR/ABL+ CML in chronic phase

o Documented chronic phase CML must meet all the following criteria:

< 15% blasts in peripheral blood

< 30% blasts plus promyelocytes in peripheral blood

< 20% basophils in the peripheral blood

≥ 100 x 109/L (≥ 100,000/mm3) platelets

- Age ≥18

- ECOG performance status of 0-2

- Evidence of typical BCR-ABL transcripts which are amenable to standardized RQ-PCR

- Adequate end organ function as defined by:

o Total bilirubin < 1.5 x ULN (ULN = upper limit of normal in a local institution lab).

Does not apply to patients with isolated hyperbilirubinemia (e.g., Gilbert’s disease) grade < 3

o SGOT (AST) and SGPT (ALT) ≤ 3 x ULN

o Serum amylase and lipase ≤ 2 x ULN

o Alkaline phosphatase ≤ 2.5 x ULN

o Serum creatinine < 1.5 x ULN

- Written informed consent prior to any study procedures.

**Exclusion criteria**

Previous treatment with BCR-ABL inhibitors for more than 30 days.

Expression of any atypical BCR-ABL transcripts, instead of the classical P210-encoding type with the e13a2 or the e14a2 junction at screening.

Previous anticancer agents (hydroxyurea, anagrelide, interferon) for CML for more than three months.

Poorly controlled diabetes mellitus (defined as HbA1c >8%).

Prior documented history of coronary heart disease, including myocardial infarction, coronary bypass, coronary stent, and symptomatic angina.

Uncontrolled hypertension.

History of peripheral arterial occlusive disease.

History of acute pancreatitis within 12 months of study entry, or a past medical history of chronic pancreatitis.

Patients actively receiving therapy with strong CYP3A4 inhibitors and/or inducers which cannot be either discontinued or switched to a different medication prior to starting study drug.

Patients who are currently receiving treatment with any medications that have the potential to prolong the QT interval and for which cannot be either safely discontinued or switched to a different medication prior to starting study drug.

Women of childbearing potential, defined as all women physiologically capable of becoming pregnant, unless they are using effective methods of contraception during dosing of study treatment.

Patients unable to understand and to comply with study instructions and requirements.

Refusal to give informed consent.

**Sample size computation**

For the first co-primary endpoint the sample size computation considers that 25% of the patients randomized to the NIL arm are expected to reach MR4.5 at 24 months. Since the lack of data on the sequence IMA-NIL, the expected rate of MR4.5 at 24 months of the IMarm was increased to 9% from the 6% of the ENESTnd trial. Accordingly, a total of 260 patients (130 per treatment arm) are needed to detect this difference as statistically significant with 90% power and a two-sided stratified Cochran-Mantel-Haenszel test at the 3% level. The proportion of patients reaching stable MR4.0 during the consolidation period, entering the TFR phase, and free from relapse after one year is expected to be 27% and 12% of all patients randomized to NIL and IMA, respectively. Consequently, for the second co-primary endpoint of the study a total of 360 patients, 180 per treatment group, will allow a 90% power to detect the expected difference as statistically significant by a two-sided stratified Cochran-Mantel-Haenszel test at the 3% level. In conclusion, to consider a small dropout rate, a total of 450 patients (225 per treatment group) were planned to be enrolled in the study.

The two randomized treatment groups will be compared under the Intention-to-Treat (ITT) principle.

**Figure 1.** Study design.


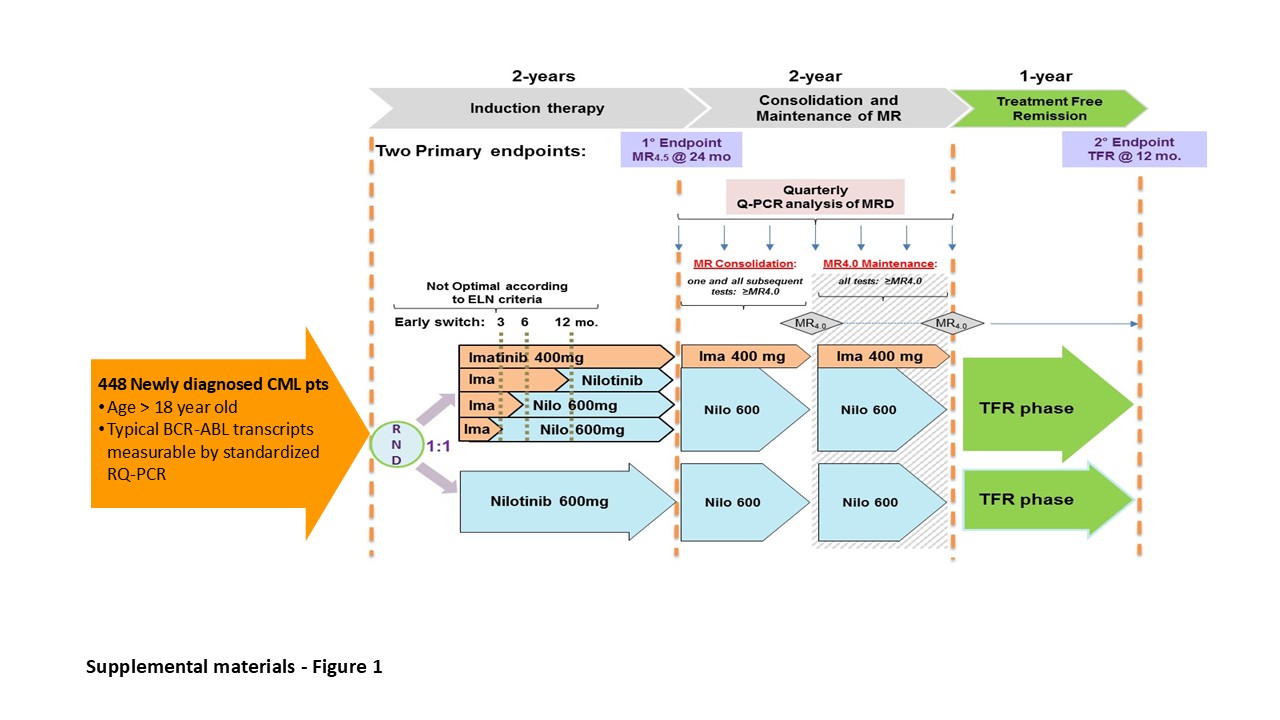

Supplement: Supplementary file 1 — Supplementary material [file 41375_2025_2796_MOESM1_ESM.docx]
